# Development of nomograms for predicting the survival of intestinal-type gastric adenocarcinoma patients after surgery

**DOI:** 10.1038/s41598-023-44671-w

**Published:** 2023-10-13

**Authors:** Chu-Yun Liu, Yu-Shen Yang, Kai Ye, He-fan He

**Affiliations:** 1 Department of Anaesthesiology, The Second Affiliated Hospital of Fujian Medical University, No. 34 North Zhongshan Road, Quanzhou, 362000 Fujian China; 2 Department of General Surgery, The Second Affiliated Hospital of Fujian Medical University, No. 34 North Zhongshan Road, Quanzhou, 362000 Fujian China

**Keywords:** Computational biology and bioinformatics, Oncology

## Abstract

Intestinal-type gastric adenocarcinoma (IGA) is a common phenotype of gastric cancer. Currently, few studies have constructed nomograms that may predict overall (OS) and cancer-specific survival (CSS) probability after surgery. This study is to establish novel nomograms for predicting the survival of IGA patients who received surgery. A total of 1814 IGA patients who received surgery between 2000 and 2018 were selected from Surveillance, Epidemiology, and End Results database and randomly assigned to the training and validating sets at a ratio of 7:3. Then univariate and multivariate cox regression analyses were performed to screen significant indictors for the construction of nomograms. The calibration curve, the area under the receiver operating characteristic (receiver operating characteristic, ROC) curve (the area under curve, AUC), C-index, net reclassification index (NRI), integrated discrimination improvement (IDI) and decision curve analysis (DCA) curves were applied to assess the performance of the model. The significant outcomes of multivariate analysis revealed that ten variables (age, sex, race, surgery type, summary stage, grade, AJCC TNM stage, radiotherapy, number of regional nodes examined, number of regional nodes positive) were demonstrated to construct the nomogram for OS and ten variables (age, sex, race, surgery type, summary stage, grade, AJCC TNM stage, chemotherapy, number of regional nodes examined, number of regional nodes positive) for CSS. The calibration and AUC uncovered their favorable predictive performance. Subsequently, C-index, NRI, IDI and DCA curves further validated the predicative superiority of nomograms over 7th AJCC Stage System. The validated nomogram provides more reliable OS and CSS predictions for postoperative IGA patients with good accuracy, which can help surgeons in treatment decision-making and prognosis evaluation.

## Introduction

As a common type of malignancy (the fifth most common cancer and the third major reason of cancer-associated death worldwide^1^), more than 25,000 new cases of gastric cancer (GC) and 11,000 fatal cases were determined in U.S. for 2019^2^. The onset of GC presents strong regional and gender features. Nearly 70% of patients with GC were diagnosed in developing countries, while the incidence of GC in male population is twice as much as female population^3^. The most prevalent type of GC is gastric adenocarcinoma^4^, which are further classified into different histologic subtypes according to the Lauren classification^5^, including diffuse (32%), intestinal (54%), and indeterminate type (15%). The initial stages of GC are usually asymptomatic and hard to be detected, so most of patients are diagnosed at advanced stages. This calls out a huge challenge which urges individualized and precise treatment for such patients.

Surgical operation brings a curative hope for the vast majority of patients and is regarded as the major foundation of holistic management of GC. Especially, the recent development of sentinel lymph node biopsy and indocyanine green fluorescence further increase the achievement ratio of stomach-sparing procedures, thus greatly improving quality of life without compromising oncological radicality^6^. However, while completely surgical resection can eliminate the visible lesions in the field of operation^7^, tumor recurrence still potentially occurs because of the residual micro-metastatic cells outside of the surgical field. Such unseen micro-metastatic cells eventually evolve into a tumor lump that can cause great suffering and burden for patients^8^. Thus, an effective way for the prognostic evaluation after surgery becomes urgently necessary.

Intestinal-type gastric adenocarcinoma (IGA) is the commonest subtype of GC^9^, which is most often diagnosed in older patients^10^. It’s reported that some ordinary environmental factors (including smoking, alcohol, diet, and H. pylori infection) are tightly associated with IGA^11^. However, the factors influencing the postoperative prognosis of IGA have not been detailly depicted yet.

Recently, the nomogram has been widely used to provide accurate evaluation for oncological disease because of its’ simple, intuitive, and practical characteristics^12,13^. In this study, we planned to use the Surveillance, Epidemiology, and End Results (SEER) database to develop more detailed nomogram to predict the survival of postoperative IGA patients. On the other hand, the tumour-lymph node-metastasis (TNM) stage system on basis of the anatomic extent of tumour is released by American Joint Committee on Cancer (AJCC) and have always been the benchmark in clinical practice around the world. Thus, the predication performance of our nomograms was evaluated by comparison with AJCC TNM stage system.

## Methods

### Patient selection

**Figure 1:**
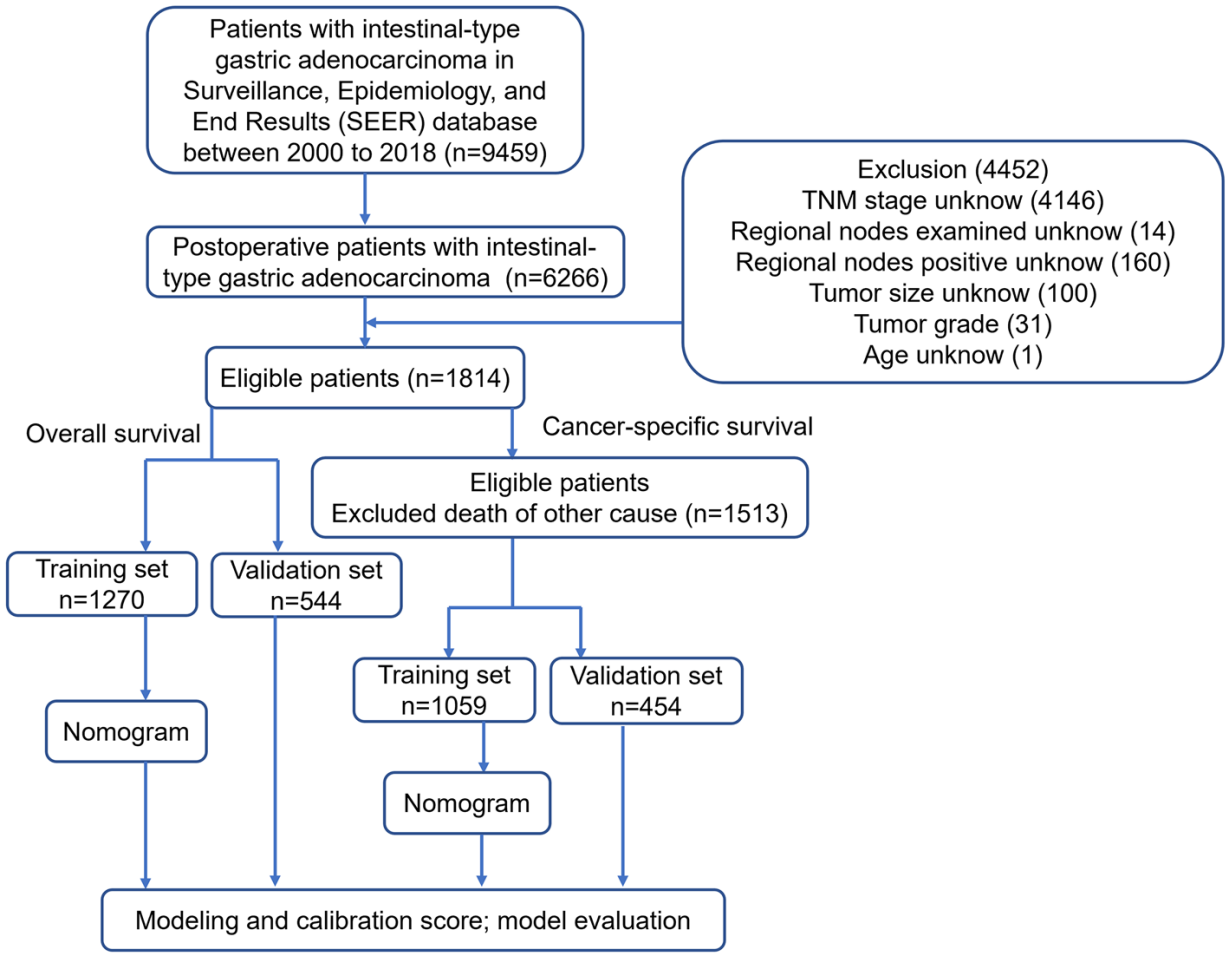
Flow diagram of patients’ screen.

The data of IGA patients (between 2000 and 2018) was screened from SEER 18 registries database (with additional treatment fields) using SEER ∗ Stat software (version. 8.4.0). As the Data Use Agreement to the SEER Program has been signed by us, we were allowed to access the SEER data without the need to apply for local ethical approval or declaration. The data utilized for the current study was extracted according to strict inclusion and exclusion criteria. The information of total 9459 patients were obtained following SEER variables: age at diagnosis, sex, race, marital status, surgery type, tumor grade, primary site, summary stage, AJCC TNM stage, chemotherapy, radiation therapy, the number of regional nodes examined (RNE) and regional nodes positive (RNP), tumor size and survival time. The exclusion criteria were listed below: (1) cases with non-surgical treatment or unknow, (2) unknow AJCC TNM stage at diagnosis, (3) unclear characteristic data. Figure 1 displayed the flowchart of data screen.

### Data collection, construction and validation of the nomogram

The included IGA patients were randomly divided into training and validation cohorts at a ratio of 7:3 using completely randomized digital table. The training set was used to establish the nomogram, and then the validation set was chosen to optimize and evaluate the model parameters. In this study, we extracted 16 clinicopathological factors from the SEER database: age, sex (male and female), race (white; black; other), marital status (single; married; other), surgery type (local tumor excision; partial/subtotal/hemi-gastrectomy; near total or total gastrectomy; gastrectomy with removal of a portion of esophagus; gastrectomy with a resection in continuity with the resection of other organs; other), primary site (fundus; cardia; body; lesser; greater; gastric antrum; pylorus; other), grade (grade I; grade II; grade III; and grade IV), summary stage (localized; regional; and distant), 7th AJCC stage (I; II; III; IV), T stage (T1; T2; T3; and T4), N stage (N0; N1; N2 and N3), M stage (M0; M1), radiotherapy (yes or no), chemotherapy (yes or no), RNE and RNP, and tumor size. The follow-up data were used for overall survival (OS) and cancer-specific survival (CSS) analysis. All of thirteen prognostic factors (excluding T stage, N stage, and M stage) were included in univariate Cox regression analysis and then independent prognostic factors were obtained via multivariate Cox regression analysis based on the results of univariate Cox regression analysis (P < 0.05). Subsequently, the factors significantly associated with OS or CSS were selected to create the nomogram, while internal validation was conducted. Firstly, the performance of the nomogram was measured by calibration curves and the area under receiver operating characteristic (ROC) curve (AUC). Next, the predicative ability of the nomogram and the 7^th^ AJCC TNM stage system was compared by C-index, net reclassification index (NRI), integrated discrimination improvement (IDI) and Decision curve analysis (DCA) curves. This study was performed under the guidance of the "TRIPOD" guideline^14^.

### Statistical analysis

R software (version 4.2.1) was used to perform all statistical analyses. The two-sided P < 0.05 was set as the cutoff of significance.

## Results

### Clinical characteristics

A total of 1814 patients were included into OS analysis, which were randomly assigned training (n = 1270) and validation (n = 544) cohorts. The clinical characteristics of IGA patients were described below. In the training set, the median age at diagnosis was 72 years (range 18–98 years). There were 453 (35.7) female patients and 817 (64.3) male ones, among which white people accounted for 56.6%, while black people for 16.4% and other races for 27.0%. These patients were majorly married (59.1%), while 161 of them were single (12.7%) and 359 were of other marital status (28.3%). More than half these patients (62.6%) received near total or total gastrectomy. The primary sites were located in cardia/fundus (14.7%), gastric body (30.9%), gastric pylori (39.3%), and other part of stomach (15.1%). Moderate (47.4%) and worse (42.1%) differentiation were the commonest tumor grades, followed by well differentiation (9.5%) and undifferentiation (0.9%). The summary stage of IGA consisted of localized (40.9%), regional (46.9%) and distant cancer (12.2%). Most patients (33.3%) were clarified into stage I, 31.2% were stage III, 27.1% were stage II, and 8.4% were stage IV. Only 24.8% had the radiation record, while almost half of the patients (43.1%) had the chemotherapy record. The median RNE was 17 (range 1–76), while the median RNP was 1 (range 0–44) and median tumor size was 40 (range 1–165). In the validation set, patients displayed similar characteristics to those in the training cohort. On the other hand, there were 1513 patients in the study for CSS analysis with 1059 patients in the training set and the remaining 454 patients in the validation set. Both sets in CSS group also shared similar clinical characteristics. Table 1 summarized the clinic-pathological characteristics of patients in the OS and CSS groups.

**Table 1:** Patient characteristics in the study.

Variables	OS group (n = 1814)		CSS group (n = 1513)	
	Training cohort (n = 1270)	Validation cohort (n = 544)	Training cohort (n = 1059)	Validation cohort (n = 454)
Age (years)				
Median	72	73	72	72
Range	18–98	29–98	21–96	29–97
Sex				
Female	453 (35.7)	184 (33.8)	385 (36.4)	155 (34.1)
Male	817 (64.3)	360 (66.2)	674 (63.6)	299 (65.9)
Marital status				
Single	161 (12.7)	75 (13.8)	148 (14.0)	50 (11.0)
Married	750 (59.1)	322 (59.2)	608 (57.4)	301 (66.3)
Other	359 (28.3)	147 (27.0)	303 (28.6)	103 (22.7)
Race				
White	719 (56.6)	304 (55.9)	591 (55.8)	253 (55.7)
Black	208 (16.4)	88 (16.2)	175 (16.5)	66 (14.5)
Other	343 (27.0)	152 (27.9)	293 (27.7)	135 (29.7)
Surgical type				
Local tumor excision	4 (0.3)	2 (0.4)	4 (0.4)	1 (0.2)
^a^Gastrectomy	795 (62.6)	351 (64.5)	663 (62.6)	288 (63.4)
^b^Gastrectomy	184 (14.5)	67 (12.3)	159 (15)	56 (12.3)
^c^Gastrectomy	144 (11.3)	64 (11.8)	116 (11)	60 (13.2)
^d^Gastrectomy	140 (11)	58 (10.7)	115 (10.9)	47 (10.4)
Other	3 (0.2)	2 (0.4)	2 (0.2)	2 (0.4)
Primary site				
Cardia/Fundus	187 (14.7)	75 (13.8)	152 (14.4)	71 (15.6)
Body	392 (30.9)	157 (28.9)	302 (28.5)	143 (31.5)
Pylori	499 (39.3)	240 (44.1)	453 (42.8)	167 (36.8)
Other	192 (15.1)	72 (13.2)	152 (14.4)	73 (16.1)
Grade				
I	121 (9.5)	67 (12.3)	112 (10.6)	49 (10.8)
II	602 (47.4)	263 (48.3)	504 (47.6)	211 (46.5)
III	535 (42.1)	207 (38.1)	433 (40.9)	187 (41.2)
IV	12 (0.9)	7 (1.3)	10 (0.9)	7 (1.5)
Summary stage				
Localized	520 (40.9)	235 (43.2)	425 (40.1)	182 (40.1)
Regional	595 (46.9)	257 (47.2)	516 (48.7)	210 (46.3)
Distant	155 (12.2)	52 (9.6)	118 (11.1)	62 (13.7)
AJCC stage				
I	423 (33.3)	185 (34.0)	349 (33.0)	145 (31.9)
II	344 (27.1)	173 (31.8)	292 (27.7)	119 (26.2)
III	396 (31.2)	145 (26.7)	333 (31.4)	148 (32.6)
IV	107 (8.4)	41 (7.5)	85 (8)	42 (9.3)
Radiotherapy				
No	955 (75.2)	415 (76.3)	794 (75)	335 (73.8)
Yes	315 (24.8)	129 (23.7)	265 (25)	119 (26.2)
Chemotherapy				
No	723 (56.9)	314 (57.7)	577 (54.5)	249 (54.8)
Yes	547 (43.1)	230 (42.3)	482 (45.5)	205 (45.2)
RNE				
Median	17	15	16	17
Range	1–76	1–71	1–76	1–64
RNP				
Median	1	0.5	1	1
Range	0–44	0–33	0–44	1–40
Tumor size (mm)				
Median	40	41.5	40	40
Range	1–165	1–160	1–165	3–120

^a^Partial, subtotal, hemi-Gastrectomy.

^b^Near total or total gastrectomy.

^c^Gastrectomy with removal of a portion of esophagus.

^d^Gastrectomy with a resection in continuity with the resection of other organs.

### Nomogram construction

**Figure 2:**
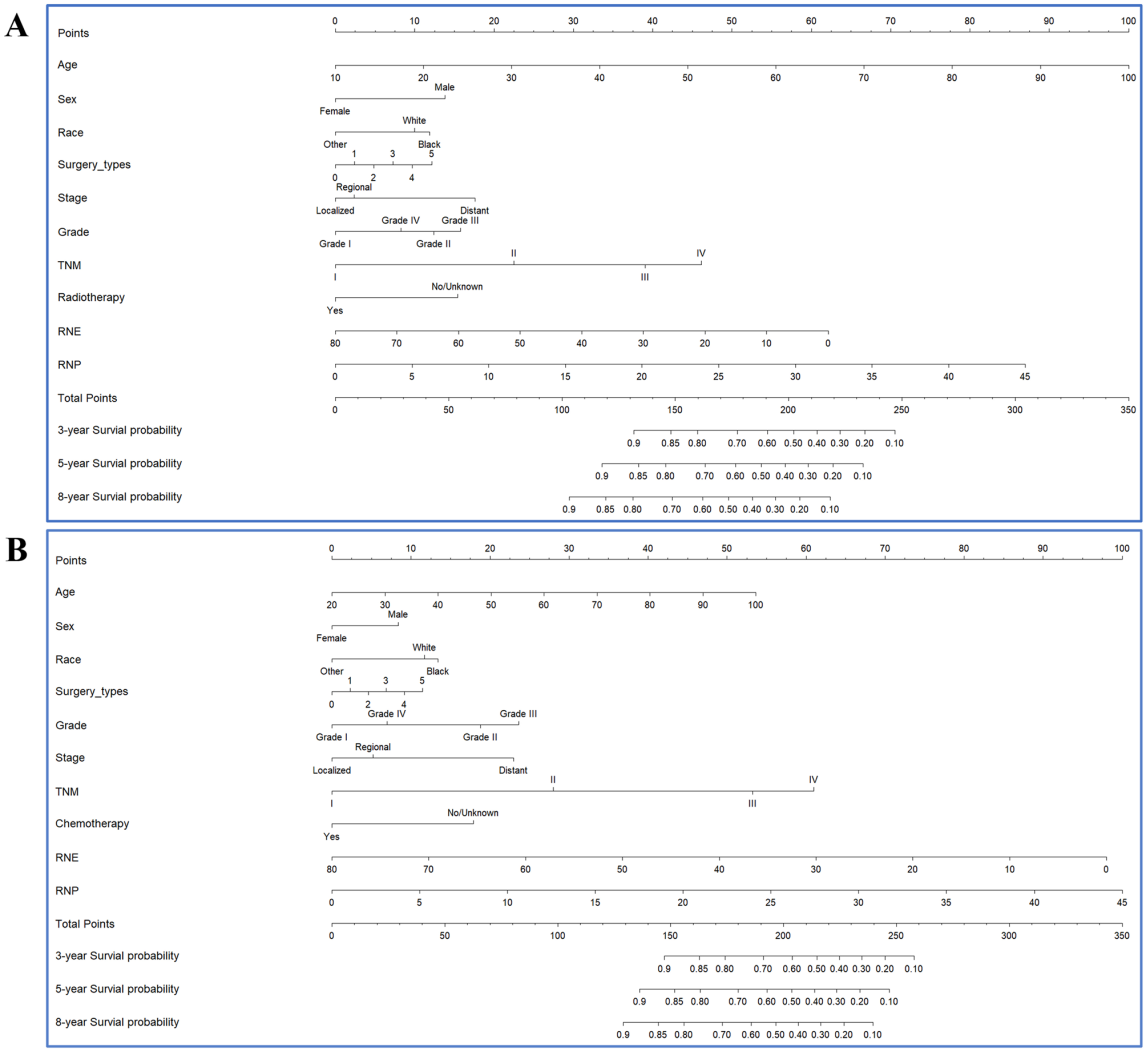
Nomograms predicting 3-, 5- and 8-year OS (**A**) and CSS (**B**) of postoperative IGA patients. Note: 0, local tumor excision; 1, partial/subtotal/ hemi-gastrectomy; 2, near total or total gastrectomy; 3, gastrectomy with removal of a portion of esophagus; 4, gastrectomy with a resection in continuity with the resection of other organs; 5, other.

Two nomograms for IGA patients who receive surgery were established based on the variables screen from Cox analysis. After the multivariable Cox analysis, the outcomes in OS group revealed that the age at diagnosis, sex, race, surgery type, summary stage, grade, AJCC TNM stage, radiotherapy status, RNE and RNP can independently predict the OS of IGA patients who receive surgery (Table 2). In CSS group, the results from the multivariable Cox analysis demonstrated that the age at diagnosis, sex, race, surgery type, summary stage, grade, AJCC TNM stage, chemotherapy status, RNE and RNP are the independent risk factors of CSS for IGA patients who receive surgery (Table 3). All of these independent factors that were associated with OS and CSS were included in the prognostic nomogram created in this study (Fig. 2).

**Table 2:** Univariate and multivariate analysis of overall survival with IGA.

Variables	Univariate analysis		Multivariate analysis	
	HR (95% CI)	*P*	HR (95% CI)	*P*
Age (years)	1.023 (1.016–1.03)	8.4e − 11	1.03 (1.022 ~ 1.037)	6.70E − 16
Sex				
Female	1.284 (1.098–1.502)	0.002	Reference	
Male			1.45 (1.231 ~ 1.708)	8.40E − 06
Marital status				
Single	1.104 (0.9796–1.243)	0.105		
Married				
Other				
Race				
White	0.856 (0.784–0.934)	< 0.001	1.282 (1.072 ~ 1.533)	6.50E − 03
Black			1.33 (1.042 ~ 1.698)	2.20E − 02
Other			Reference	
Surgical methods				
Local tumor excision	1.083 (1.012–1.158)	0.020	1.984 (0.863 ~ 4.562)	1.10E − 01
^a^Gastrectomy			Reference	
^b^Gastrectomy			1.234 (0.974 ~ 1.562)	8.20E − 02
^c^Gastrectomy			1.109 (0.874 ~ 1.408)	4.00E − 01
^d^Gastrectomy			1.173 (0.929 ~ 1.482)	1.80E − 01
Uncertain surgery			3.988 (2.208 ~ 7.203)	4.50E − 06
Primary site				
Cardia/Fundus	0.9786 (0.9026–1.061)	0.599		
Body				
Pylori				
Other				
Grade				
I	1.408 (1.257–1.578)	3.4e − 09	Reference	
II			1.413 (0.995 ~ 2.006)	5.30E − 02
III			1.532 (1.065 ~ 2.202)	2.10E − 02
IV			1.241 (0.563 ~ 2.735)	5.90E − 01
Summary stage				
Localized	2.146 (1.925–2.393)	< 2e − 16	Reference	
Regional			1.076 (0.837 ~ 1.384)	5.70E − 01
Distant			1.636 (1.107 ~ 2.419)	1.40E − 02
AJCC stage				
I	1.83 (1.694–1.978)	< 2e − 16	Reference	
II			1.743 (1.334 ~ 2.277)	4.70E − 05
III			2.646 (1.893 ~ 3.698)	1.20E − 08
IV			3.16 (1.986 ~ 5.028)	1.20E − 06
Radiotherapy				
No	0.8211 (0.691–0.976)	0.025	Reference	
Yes			0.675 (0.56 ~ 0.812)	3.30E − 05
Chemotherapy				
No	0.964 (0.8314–1.118)	0.627		
Yes				
RNE	0.9922 (0.986–0.998)	0.011	0.979 (0.972 ~ 0.985)	4.00E − 10
RNP	1.059 (1.049–1.069)	< 2e − 16	1.052 (1.035 ~ 1.069)	3.00E − 10
Tumor size	1.011 (1.008–1.013)	< 2e − 16	1.002 (0.999 ~ 1.005)	2.70E − 01

*HR* hazard ratio, *RNE* number of regional nodes examined, *RNP* number of regional nodes positive, *AJCC* American-Joint Committee on Cancer.

^a^Partial, subtotal, hemi-Gastrectomy.

^b^Near-total or total gastrectomy.

^c^Gastrectomy with removal of a portion of esophagus.

^d^Gastrectomy with a resection in continuity with the resection of other organs; Univariate analysis, Kaplan–Meier analysis; multivariate analysis, cox regression analysis.

**Table 3:** Univariate and multivariate analysis of cancer-specific survival with IGA.

Variables	Univariate analysis		Multivariate analysis	
	HR (95% CI)	*P*	HR (95% CI)	*P*
Age (years)	1.017 (1.009 ~ 1.025)	4.83E − 05	1.019 (1.011 ~ 1.028)	< 0.001
Sex				
Female	1.214 (1.007 ~ 1.465)	0.0423	Reference	
Male			1.272 (1.047 ~ 1.547)	0.016
Marital status				
Single	1.087 (0.945 ~ 1.25)	0.244		
Married				
Other				
Race				
White	0.821 (0.739 ~ 0.912)	< 0.001	1.362 (1.093 ~ 1.698)	0.006
Black			1.439 (1.087 ~ 1.904)	0.011
Other			Reference	
Surgical methods				
Local tumor excision	1.108 (1.022 ~ 1.200)	0.012	1.574 (0.965 ~ 2.567)	0.069
^a^Gastrectomy			Reference	
^b^Gastrectomy			1.372 (1.053 ~ 1.788)	0.019
^c^Gastrectomy			1.133 (0.846 ~ 1.518)	0.400
^d^Gastrectomy			1.150 (0.866 ~ 1.525)	0.330
Uncertain surgery			2.390 (0.732 ~ 7.802)	0.150
Primary site				
Cardia/Fundus	0.934 (0.846 ~ 1.030)	0.172		
Body				
Pylori				
Other				
Grade				
I	1.510 (1.318 ~ 1.730)	3.01E − 09	Reference	
II			1.725 (1.076 ~ 2.767)	0.024
III			1.941 (1.197 ~ 3.146)	0.007
IV			1.170 (0.510 ~ 2.681)	0.710
Summary stage				
Localized	2.663 (2.328 ~ 3.045)	< 2e − 16	Reference	
Regional			1.168 (0.819 ~ 1.666)	0.390
Distant			1.907 (1.202 ~ 3.025)	0.006
AJCC stage				
I	2.240 (2.032 ~ 2.469)	< 2e − 16	Reference	
II			2.039 (1.378 ~ 3.017)	< 0.001
III			3.991 (2.505 ~ 6.359)	5.8E − 09
IV			5.012 (2.816 ~ 8.919)	4.3E − 08
Radiotherapy				
No	1.029 (0.843 ~ 1.256)	0.778		
Yes				
Chemotherapy				
No	1.207 (1.011 ~ 1.44)	0.037	Reference	
Yes			0.618 (0.497 ~ 0.769)	< 0.001
RNE	0.988 (0.981 ~ 0.996)	0.002	0.966 (0.957 ~ 0.974)	2.4E − 14
RNP	1.075 (1.064 ~ 1.086)	< 2e − 16	1.064 (1.046 ~ 1.082)	6.2E − 13
Size	1.011 (1.009 ~ 1.014)	5.8E − 16	1.002 (0.998 ~ 1.006)	0.290

*HR* hazard ratio, *RNE* number of regional nodes examined, *RNP* number of regional nodes positive, *AJCC* American-Joint Committee on Cancer.

^a^Partial, subtotal, hemi-Gastrectomy.

^b^Near-total or total gastrectomy.

^c^Gastrectomy with removal of a portion of esophagus.

^d^Gastrectomy with a resection in continuity with the resection of other organs; Univariate analysis, Kaplan–Meier analysis; multivariate analysis, cox regression analysis.

### Validation of the nomogram for OS and CSS of IGA patients who receive surgery

**Figure 3:**
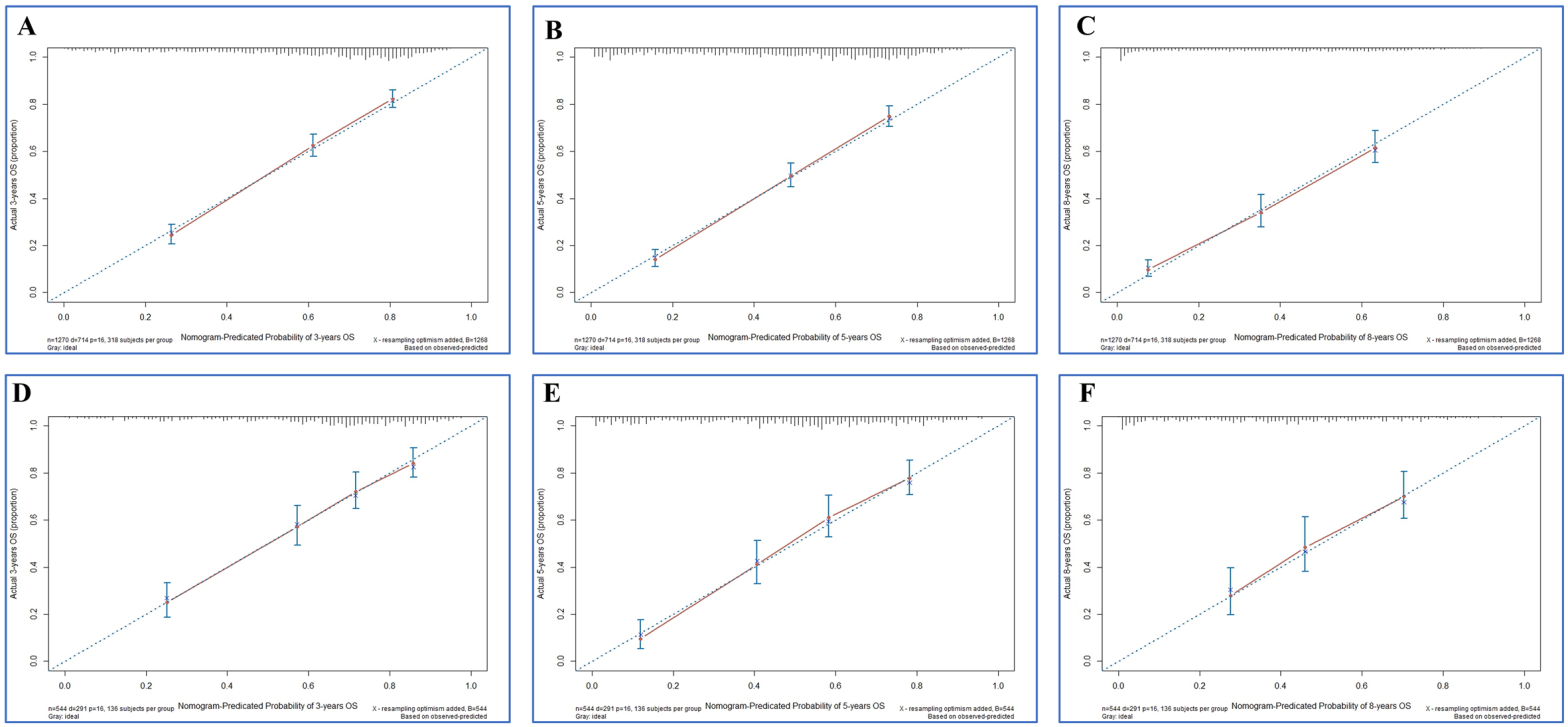
Calibration plots of the nomogram for 3-, 5- and 8-year OS prediction of the training cohort (**A**–**C**) and internal validation cohort (**D**–**F**).

**Figure 4:**
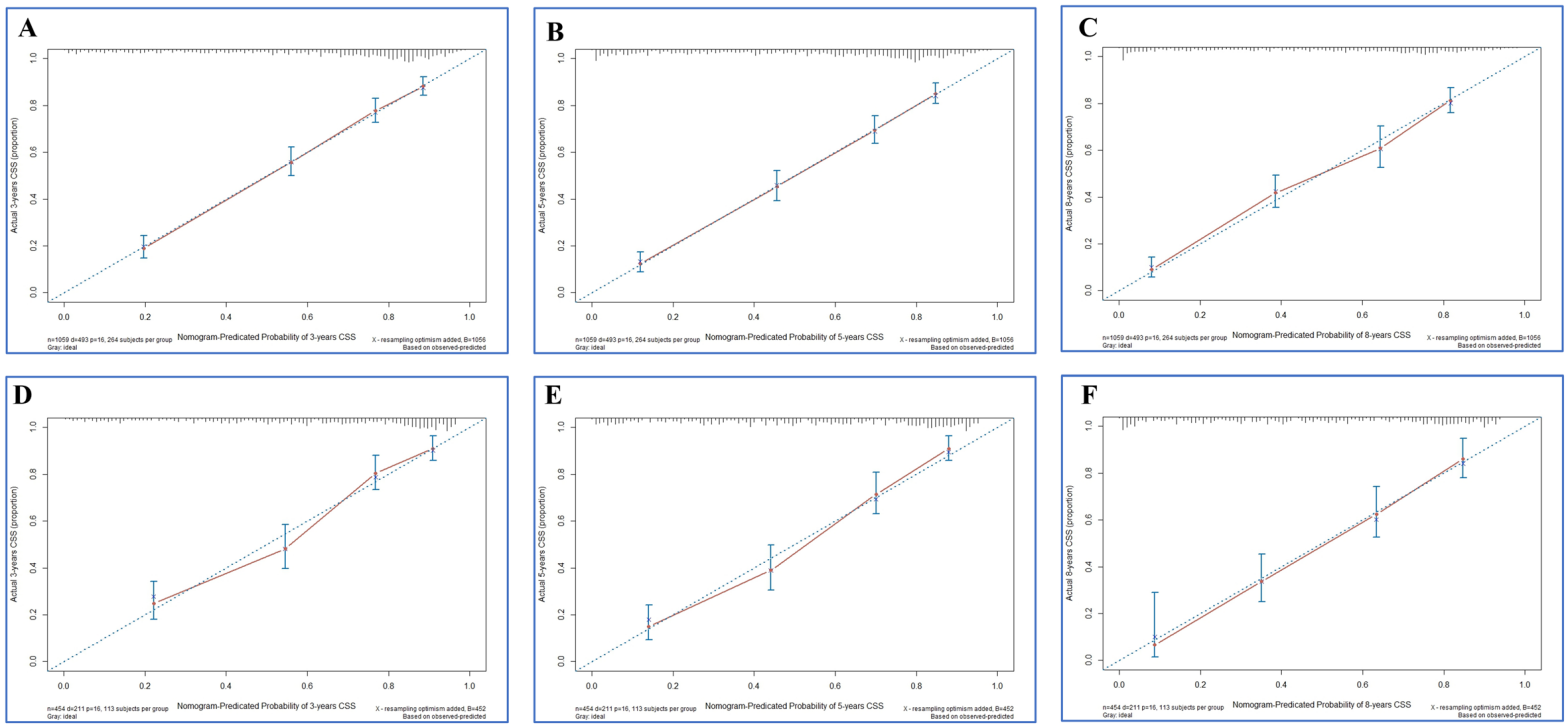
Calibration plots of the nomogram for 3-, 5- and 8-year CSS prediction of the training cohort (**A**–**C**) and internal validation cohort (**D**–**F**).

**Figure 5:**
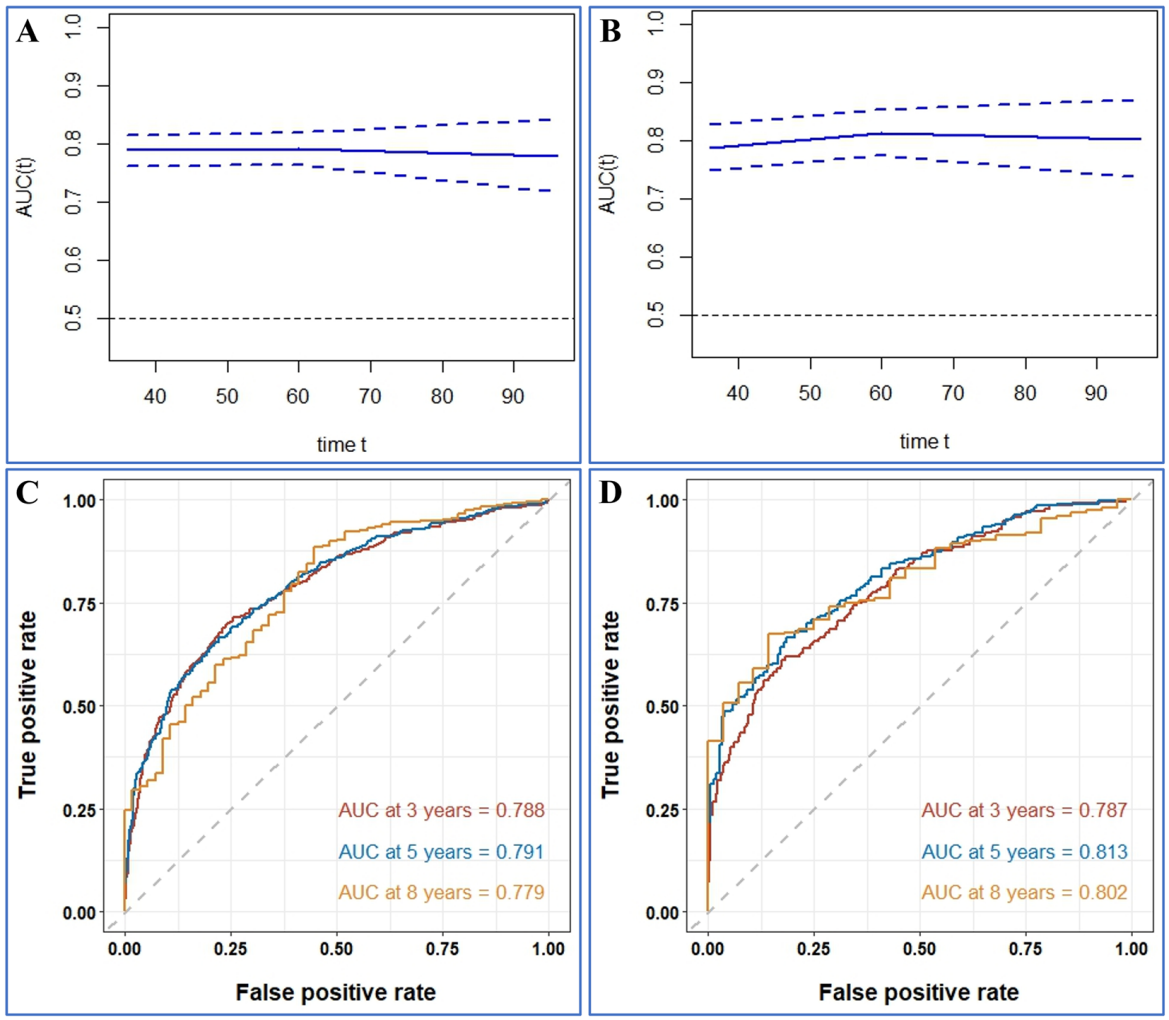
Time-dependent AUC and receiver operating characteristic (ROC) curves of OS. (**A**,**B**) Time-dependent AUC of using the nomogram to OS probability within 8 years in the training cohort and validation cohort. The shading area between blue dotted curves represents 95% credible intervals. (**C**,**D**) ROC curves corresponding to 1-, 3-, and 8-year OS in the training and validation cohort, respectively.

**Figure 6:**
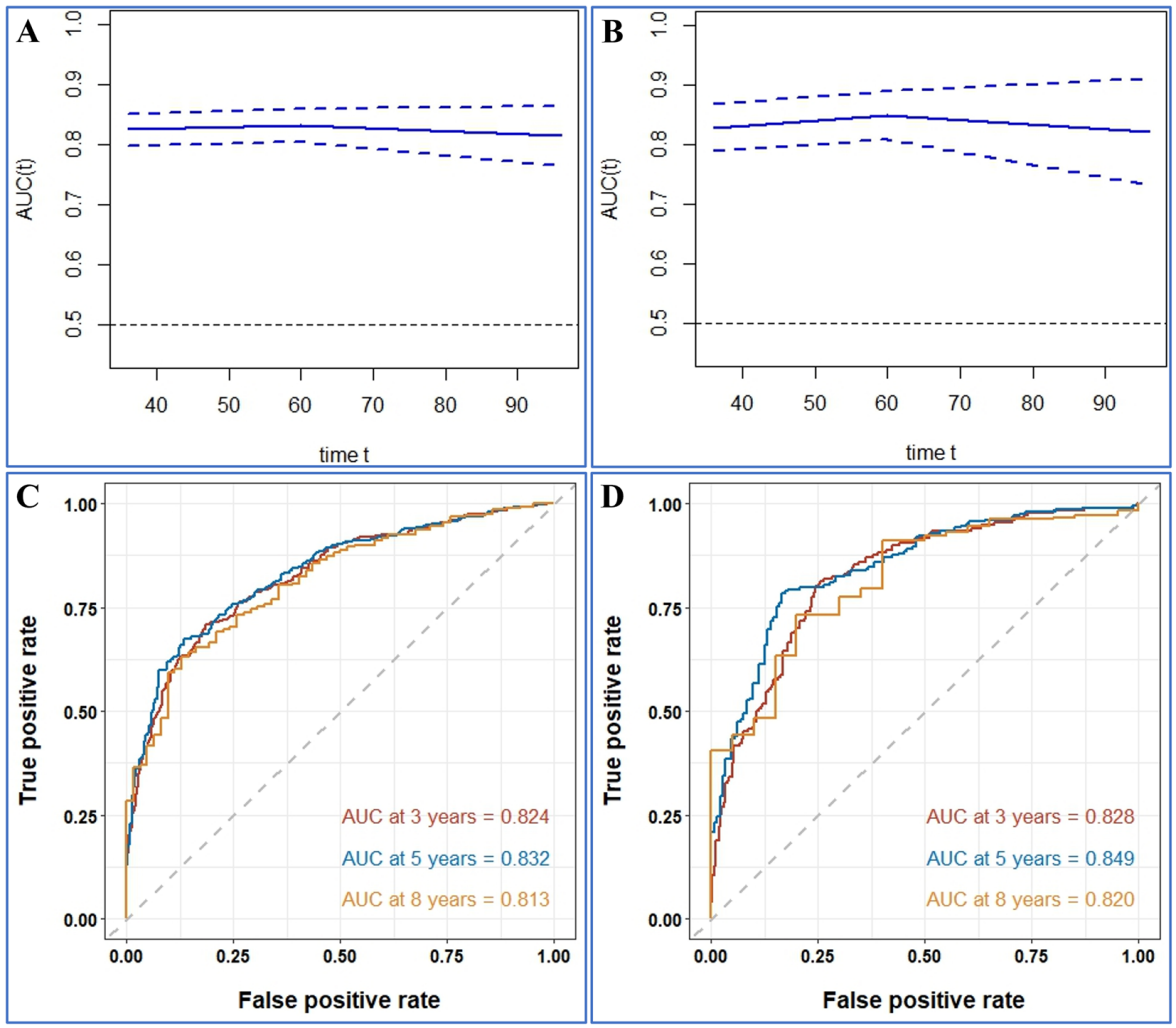
Time-dependent AUC and receiver operating characteristic (ROC) curves of CSS. (**A**,**B**) Time-dependent AUC of using the nomogram to CSS probability within 8 years in the training cohort and validation cohort. The shading area between blue dotted curves represents 95% credible intervals. (**C**,**D**) ROC curves corresponding to 1-, 3-, and 8-year CSS in the training and validation cohort, respectively.

Firstly, the calibration curves of these nomograms were established in OS and CSS group and the results displayed almost identical consistency of the actual likelihood with the predicted 3-, 5-, and 8-year probabilities in the training and validation set (Figs. 3, 4). Next, the results from the time-dependent AUC curves in the Cox models of OS and CSS group confirmed that AUCs were almost greater than 0.7 for the forecast of OS and CSS within eight years, suggesting the nomogram to be good discriminative ability (Figs. 5A,B and 6A,B). In addition, the AUCs of OS group in the training set, for predicting 1, 3, and 8 years were 0.788, 0.791, and 0.779, respectively (Fig. 5C). In the validation set, the AUCs at 1, 3, and 8 years were 0.787 0.813, and 0.802, respectively (Fig. 5D). Furthermore, the AUCs of the CSS group in the training cohort were 0.824 at 3 years, 0.832 at 5 years and 0.813 at 8 years, while in the validation cohort the AUCs were 0.828 at 3 years, 0.849 at five years and 0.820 at eight year (Fig. 6C,D).

### Comparison of the values between nomograms and AJCC stage system

**Figure 7:**
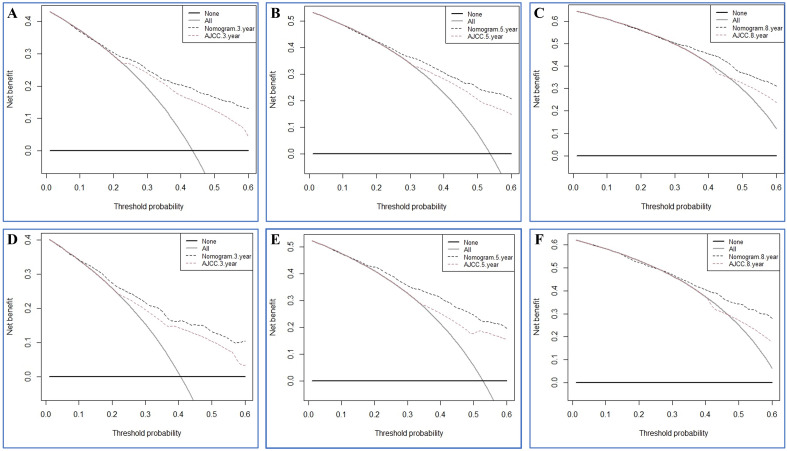
Decision curve analysis of the nomogram in the estimation of OS of postoperative IGA patients. (**A**–**C**) Training cohort. (**D**–**F**) Validation cohort.

**Figure 8:**
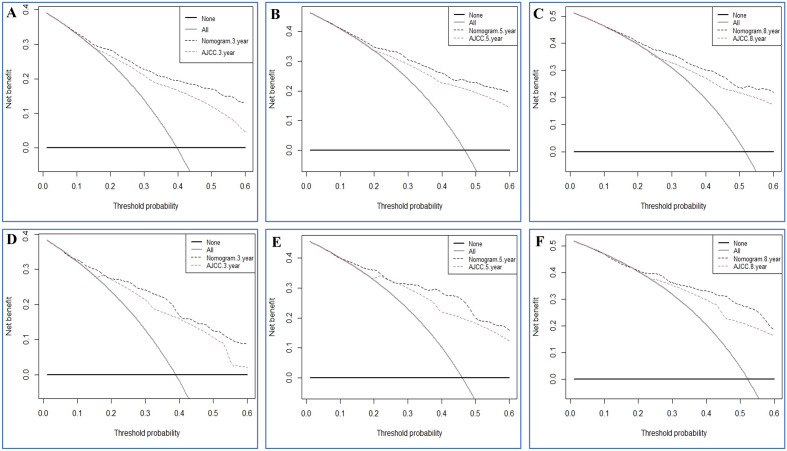
Decision curve analysis of the nomogram in the estimation of CSS of postoperative IGA patients. (**A**–**C**) Training cohort. (**D**–**F**) Validation cohort.

In order to further evaluate the clinical performance of our nomograms, their predictive capacity was directly contrast 7th AJCC TNM staging system for IGA following surgery. In OS group, the C-indexes for the nomogram in the training and validation sets (0.785 and 0.802, respectively) were larger compared to the 7th AJCC staging system (0.704 and 0.690). In CSS group, the nomogram of the training and validation cohorts (0.819 and 0.838, respectively) also had higher C-index than the 7^th^ AJCC staging system (0.754 and 0.767). The NRI in the training set for the 3- , 5- and 8-year OS were 0.4882 (95% CI 0.3674–0.5965), 0.5262 (95% CI 0.4191–0.6501) and 0.5388 (95% CI 0.3878–0.7270), and the IDI values for the 3- , 5- and 8-year OS were 0.093 (95% CI 0.071–0.121, P < 0.001), 0.096 (95% CI 0.074–0.126, P < 0.001) and 0.130 (95% CI 0.074–0.181, P < 0.001) (Table 4). The outcomes of NRI and IDI from the contrasts between nomogram of CSS group and AJCC staging system similarly showed statistical significance (Table 5). Finally, the DCA analysis was performed to evaluate the 3-, 5-, and 8-year OS and CSS discrimination ability and the results are displayed in Figs. 7 and 8. The DCA plots showed good net benefits. All of these results were demonstrated in the validation set (Table 5), verifying the better predictive ability of our nomograms than the AJCC Stage System.

**Table 4:** Comparison of different models for estimating the overall survival of IGA patients.

Index	Training cohort			Validation cohort		
	Estimate	95%CI	P value	Estimate	95%CI	P value
NRI (vs. AJCC stage system)						
For 3-year OS	0.4882	0.3674–0.5965		0.5705	0.3810–0.7206	
For 5-year OS	0.5262	0.4191–0.6501		0.6779	0.4393–0.8873	
For 8-year OS	0.5388	0.3878–0.7270		0.6356	0.4191–0.9018	
IDI (vs. AJCC stage system)						
For 3-year OS	0.093	0.071–0.121	< 0.001	0.096	0.063–0.145	< 0.001
For 5-year OS	0.096	0.074–0.126	< 0.001	0.119	0.079–0.178	< 0.001
For 8-year OS	0.130	0.074–0.181	< 0.001	0.108	0.058–0.178	0.01

**Table 5:** Comparison of different models for estimating the cancer-specific survival of IGA patients.

Index	Training cohort			Validation cohort		
	Estimate	95%CI	P value	Estimate	95%CI	P value
NRI (vs. AJCC stage system)						
For 3-year CSS	0.5052	0.4015–0.6674		0.6088	0.4225–0.8043	
For 5-year CSS	0.4177	0.3086–0.5721		0.6274	0.4223–0.8192	
For 8-year CSS	0.3680	0.2523–0.5521		0.6088	0.3992–0.8405	
IDI (vs. AJCC stage system)						
For 3-year CSS	0.096	0.071–0.128	< 0.001	0.108	0.068–0.164	< 0.001
For 5-year CSS	0.095	0.067–0.126	< 0.001	0.110	0.076–0.167	< 0.001
For 8-year CSS	0.097	0.055–0.137	< 0.001	0.114	0.070–0.183	< 0.001

## Discussion

IGA is the most prevalent type of GC, which is obviously different in epidemiology, pathogenesis, prognosis, microscopic and gross appearance, and molecular characteristics from other subtypes (diffuse and intermediate type)^15^. For example, the incidence of diffuse-type GC was relatively higher in female and younger patients^16^. Importantly, a latest report indicated that for early-onset early-stage GC (diagnosed at < 50 years and limited to the mucosa or submucosa), the intestinal type showed more tight association with lymph node metastasis and worse prognosis compared to the diffuse type^17^. Thus, IGA is probably more noteworthy than other subtypes because of its’ higher incidence and worse prognosis. Up to now, the AJCC staging system is the widely accepted program for forecasting the prognosis of GC patients^18^. However, many crucial risk factors influence the OS and CSS of GC patients who received surgery as well, including age, sex, marital status, race, surgery type, primary tumor site, grade, summary stage, chemoradiotherapy and tumor size. Consequently, we constructed two nomograms to forecast the 3-, 5-, and 8-year OS and CSS of IGA patients who received surgery using the multi-center, multi-population, multi-ethnic data from the SEER database.

Our nomograms combined the AJCC staging system with basic demographics and other important oncology parameters. For all we know, these nomograms might be the first prognostic model for predicting the long-term OS and CSS (5 and 8 years) for postoperative IGA patients. In 2020, Chu et al. demonstrated that radiotherapy effectively improved the survival of patients with IGA via a SEER population‑based study^19^. In 2021, Tang et al. compared the difference of lymph node metastasis and prognosis between IGA and diffuse-type GC by screening SEER database as well^17^. Nevertheless, both studies did not try to construct a nomogram for the predication of prognosis of IGA patients. In the study, we used validation set of postoperative IGA patients from the same database to demonstrate the current nomograms. The results indicated that reliable nomograms for forecasting the 3-, 5-, and 8-year OS and CSS of postoperative IGA patients were successfully established based on good performance of nomogram validation in discriminative ability and calibration.

Several independent risk factors were incorporated into the established nomogram. The age at diagnosis is regarded as an important risk factor for prognosis of cancer patients, with survival being poorer in older patients^20,21^. The present study observed that the OS and CSS of postoperative IGA patients were negatively associated with age. Moreover, multivariate Cox analyses suggested that RNP and sex were statistical independent prognostic factors for the OS and CSS of postoperative IGA patients, and male patients had worse prognosis compared with female ones. According to the previous reports, race was tightly correlated with survival outcomes of GC patients. The black and white patients were indicated to have poorer prognosis than other races^22^. We also found that non-white or black seem to be a protective factor when compared to white or black. Currently, surgery is the only proven effective therapy for GC and intimately related to the prognosis of GC patients. This study revealed the association of the extent of surgical depletion with the prognosis of IGA patients. Furthermore, a higher AJCC stage was correlated with a worse OS and CSS, and compared with a distant summary stage, a localized summary stage was a protective factor for OS and CSS. Significantly, tumor differentiation degree was demonstrated to be associated with survival, and grade IV tumor (undifferentiated adenocarcinoma) was a risk factor for GC according to the precious results^23^. However, our multiple Cox analysis found that grade IV tumor (undifferentiated adenocarcinoma) was not obviously related to the OS and CSS in postoperative IGA patients. Dong and colleagues also obtained the same contradictory results^24^. We speculated that the small size of included patients with grade IV tumor may contribute to the conflicting phenomenon.

Other expected and noteworthy factors is radiotherapy and chemotherapy. Currently, an increasing number of studies demonstrated the role of radiotherapy in the treatment of GC patients. In the investigation of the effect of surgery plus postoperative chemoradiotherapy on the prognosis of R0 resected GC patients, Macdonald et al. found that postoperative chemoradiotherapy can prolong the median OS (from 27 to 36 months)^25^. Moreover, Shridhar et al. used the SEER database to investigate the effect of radiation and/or surgery on OS of patients with metastatic GC^26^. They demonstrated that radiation was correlated with prolonged OS in metastatic GC patients treated with surgery. Chu et al. also validated the benefits of radiation on the survival of IGA patients in a SEER population‑based study^19^. In the current study, we got the similar results that radiotherapy is an independent protective factor for OS of postoperative IGA patients. On the other hand, several clinical randomized controlled trials revealed the benefit of chemotherapy in advanced GC patients^27–29^. Importantly, Cheng et al. used their established GC database to evaluate the efficacy of oxaliplatin-based adjuvant chemotherapies in patients with distinct Lauren type GC after D2 gastrectomy^30^. Their results indicated that oxaliplatin-based adjuvant chemotherapy can obviously prolong the median disease-free survival of patients with IGA (from 18.33 months to 48.73 months). Similarly, in this predicative nomogram, we found that chemotherapy also was a statistical independent factor for CSS of postoperative IGA patients.

The included risk factors in our constructed nomograms are readily available in clinical historical records. In order to validate the accuracy of the predictive nomograms, calibration and time-dependent AUC curves were depicted. In our nomogram models, the AUC values were high (> 0.7), confirming the good discriminative ability of the models. Furthermore, we calculated and depicted the C-index, IDI, NRI, and DCA to further estimate whether the prognostic nomograms outperformed the traditional AJCC staging system. The C-index of our constructed nomograms was better than those of the AJCC staging system, demonstrating their good discrimination ability. The IDI and NRI are two more sensitive and precise indicators compared with C-index and AUC and their results reinforces the conclusion above. The IDI verified the preferably discriminative ability of the predicative models than the AJCC staging system, while the NRI suggested that the constructed model performed better than the AJCC staging system in terms of reclassifying the risk probabilities. The benefits of DCA have been reported by numerous precious studies^31–33^. In the training set and validation set in the current study, the 3-, 5-, and 8-year DCA curves showed larger net benefits than that of the traditional AJCC staging system.

Despite the nomogram performed well in predicting OS and CSS, some weaknesses of this study should be noticed. Firstly, the patient’s information collected from the SEER database, including specific radiotherapy and chemotherapy regimens, is insufficient, which probably influenced the obtained results. Secondly, the multivariate Cox regression analysis revealed grade IV tumor did not appear to be an important factor for prognosis, which obviously contradicted the clinical practice. Thirdly, other potentially important factors that could affect the survival of IGA (such as diet, smoking and alcohol consumption) were not included and thus the nomograms should be improved through further clinical trial. Fourthly, the AJCC 7th edition of the TNM stage system was used in our study for some reasons. It is widely known that the 7th edition has some shortcomings (e.g., it did not incorporate the pN3b category and its’ additional stage subgroups [stages IIB and IIIC] cannot improve predictive performance in stage-based prognosis) and thus had been replaced by 8th edition, so it's use cannot reflect real-world situations in clinic and will influence the accuracy of our model. Thus, the utilize of the 7th edition may influence the prediction results of the model. Finally, the lack of external validation by another real world weakens the reliability of the constructed models.

In conclusion, the results demonstrate that the construction of novel nomograms for forecasting OS and CSS in postoperative IGA patients is successful. The constructed nomograms not only have better predicative ability than that of the 7th AJCC staging system alone, but the indicators in the models are also routinely assessed and readily accessible in the real-world clinic. Therefore, the nomogram will assist clinicians in making personalized survival predictions and providing optimal treatment strategies for IGA patients.

## Data Availability

The datasets included in the current study can be obtained from the Surveillance, Epidemiology, and End Results (SEER) program online website (https://seer.cancer.gov/). The datasets are also available from the corresponding author upon reasonable request.
