# Author Correction: Development of nomograms for predicting the survival of intestinal-type gastric adenocarcinoma patients after surgery

**DOI:** 10.1038/s41598-023-46707-7

**Published:** 2023-11-07

**Authors:** Chu-Yun Liu, Yu-Shen Yang, Kai Ye, He-fan He

**Affiliations:** 1 Department of Anaesthesiology, The Second Affiliated Hospital of Fujian Medical University, No. 34 North Zhongshan Road, Quanzhou, 362000 Fujian China; 2 Department of General Surgery, The Second Affiliated Hospital of Fujian Medical University, No. 34 North Zhongshan Road, Quanzhou, 362000 Fujian China

Correction to: *Scientific Reports* 10.1038/s41598-023-44671-w, published online 13 October 2023

The original version of this Article contained an error in the order of the author names, which was incorrectly given as Yu-Shen Yang, Chu-Yun Liu, Kai Ye and He-fan He.

The original Article has been corrected.

